# Clinical Utility of a Comprehensive, Whole Genome CMA Testing Platform in Pediatrics: A Prospective Randomized Controlled Trial of Simulated Patients in Physician Practices

**DOI:** 10.1371/journal.pone.0169064

**Published:** 2016-12-30

**Authors:** John Peabody, Megan Martin, Lisa DeMaria, Jhiedon Florentino, David Paculdo, Michael Paul, Rena Vanzo, E. Robert Wassman, Trever Burgon

**Affiliations:** 1 University of California, San Francisco, CA, United States of America; 2 University of California, Los Angeles, CA, United States of America; 3 QURE Healthcare, San Francisco, CA, United States of America; 4 Lineagen, Salt Lake City, UT, United States of America; Centre Hospitalier Universitaire Vaudois, FRANCE

## Abstract

**Background:**

Developmental disorders (DD), including autism spectrum disorder (ASD) and intellectual disability (ID), are a common group of clinical manifestations caused by a variety of genetic abnormalities. Genetic testing, including chromosomal microarray (CMA), plays an important role in diagnosing these conditions, but CMA can be limited by incomplete coverage of genetic abnormalities and lack of guidance for conditions rarely seen by treating physicians.

**Methods:**

We conducted a longitudinal, randomized controlled trial investigating the impact of a higher resolution 2.8 million (MM) probe-CMA test on the quality of care delivered by practicing general pediatricians and specialists. To overcome the twin problems of finding an adequate sample size of multiple rare conditions and under/incorrect diagnoses, we used standardized simulated patients known as CPVs. Physicians, randomized into control and intervention groups, cared for the CPV pediatric patients with DD/ASD/ID. Care responses were scored against evidence-based criteria. In round one, participants could order diagnostic tests including existing CMA tests. In round two, intervention physicians could order the 2.8MM probe-CMA test. Outcome measures included overall quality of care and quality of the diagnosis and treatment plan.

**Results:**

Physicians ordering CMA testing had 5.43% (p<0.001) higher overall quality scores than those who did not. Intervention physicians ordering the 2.8MM probe-CMA test had 7.20% (p<0.001) higher overall quality scores. Use of the 2.8MM probe-CMA test led to a 10.9% (p<0.001) improvement in the diagnosis and treatment score. Introduction of the 2.8MM probe-CMA test led to significant improvements in condition-specific interventions including an 8.3% (p = 0.04) improvement in evaluation and therapy for gross motor delays caused by Hunter syndrome, a 27.5% (p = 0.03) increase in early cognitive intervention for FOXG1-related disorder, and an 18.2% (p<0.001) improvement in referrals to child neurology for Dravet syndrome.

**Conclusion:**

Physician use of the 2.8MM probe-CMA test significantly improves overall quality as well as diagnosis and treatment quality for simulated cases of pediatric DD/ASD/ID patients, and delivers additional clinical utility over existing CMA tests.

## Introduction

Approximately one in six U.S. children age three to 17-years have a reported developmental disability [1]. While clinically common, there is a remarkable number of genetic abnormalities known to be causal for developmental disorders (DD) that include autism spectrum disorder (ASD) and intellectual disability (ID). Some conditions, such as Down syndrome due to trisomy 21, are relatively straightforward when it comes to a genetic diagnosis, but others are not. Results from a number of studies, for example, suggest ASD is between 56–95% genetic in origin, [2–4] while another recent paper reported that 552 genes are implicated in ASD–a number that will increase as more genes are identified [5,6].

In recent years, these findings have led to the recommendation that genetics evaluations be incorporated into clinical practice guidelines for individuals with developmental disabilities. In 2010, the American College of Medical Genetics (ACMG) recommended chromosomal microarray (CMA) as the first-tier diagnostic genetic evaluation when investigating patients with DD, ASD or ID [7]. The American Academy of Pediatrics recommendation that CMA be considered as part of the initial diagnostic evaluation of patients with disorders of postnatal development including DD and ASD, was superseded by a formal guideline issued in 2014 clearly designating CMA as the first-line test, replacing the standard karyotype and fluorescent in situ hybridization tests for the child with intellectual disability of unknown etiology. These recommendations are mirrored by similar guidelines from the American Academy of Neurology and Society of Children Neurology, and the Academy of Child and Adolescent Psychiatry [8–10].

A confirmed genetic diagnosis means that associated life-threatening medical risks (seizures, tumors, renal disease, heart disease) can be better anticipated and managed, specific medications and therapies administered, and contraindicated treatments can be avoided with a predictability that was not possible before CMA testing [11–13]. Despite clear recognition of these new technologies and the clinical insights they provide, genetic testing likely remains underused [14–15]. This means fewer specific diagnoses are made and corresponding appropriate treatments offered.

Despite uniform opinion amongst the relevant societies as to the importance of CMA, a variety of CMA tests are currently on the market, which differ distinctly in their genomic coverage, both qualitatively and quantitatively, and the clinical interpretations which can be made from the various tests. This impacts the breadth of genetic detection and, ultimately, the clinical utility that testing offers to the patient and the provider.

We undertook a large-scale, experimental study to assess the clinical utility of CMA testing in general and a new 2.8MM probe-CMA test (FirstStep^Dx^ PLUS; Lineagen, Inc.), which was developed consistent with ACMG Guidelines on CMA design [16], through the addition of 88,435 probes of content validated to have sound clinical correlates with ASD and other ID/DD associated genes. The 2.8MM probe-CMA test has a higher overall detection rate than currently available CMA tests [17] and includes communication of test results to ordering physicians, and to families, via a comprehensive report that includes clear and actionable recommendations. The goal of this study was to determine the overall evidence-based practice changes that occur when a CMA test was ordered, as well as the specific impacts on diagnostic and treatment quality. This analysis is unique in that it overcomes the enormous practical problem of identifying and aggregating uncommon genetic conditions by using simulated patients to determine clinical utility. This approach allows us to look at the variability of clinical practice–what clinicians do—while holding constant the variability of (hard to find) patients with individually rare disorders.

## Methods

### Overview

We conducted a longitudinal randomized controlled study of practicing general and sub-specialty-trained pediatricians from across the U.S. without prior experience using the 2.8MM probe-CMA product (S1 Protocol). Using a “before and after” design, pediatricians were asked to care for online simulated patients, using Clinical Performance and Value (CPV®) vignettes via web-based interactive sessions [14, 18–20]. Physicians were randomized into one of two study arms: control and intervention. Physicians completed randomly assigned vignette cases at baseline (Round 1) and again 4 weeks later (Round 2). This study, which ran from August 2014 to January 2015, was conducted in accordance with ethical standards and approved on June 16, 2014, by the Chesapeake Institutional Review Board (IRB), Columbia, MD (S1 File). All participants provided written consent to participate. The trial was voluntarily listed in clinicaltrials.gov (Identifier: NCT02414438). Due to the brevity of the trial, the voluntary nature of the registration (studies not involving drugs, biologics, or devices are not required to register) and a clerical error, enrollment for this trial was underway before being logged in the registry. Notwithstanding, IRB approval was secured prior to conducting the study. The authors confirm that all ongoing and related trials for this intervention are registered.

### Eligibility and Selection of Physicians

**Fig 1:**
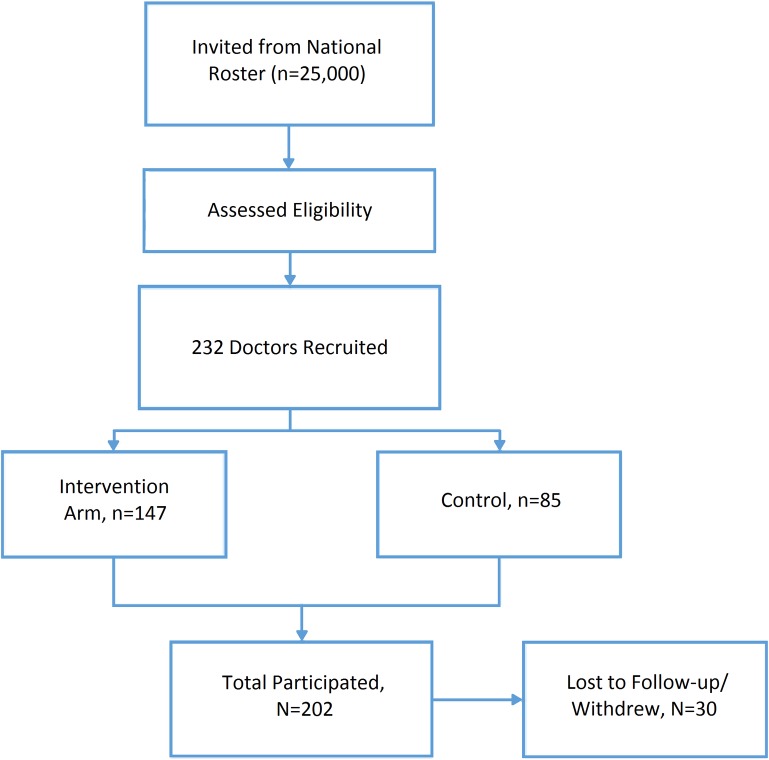
Flowchart of Physician Participant Selection. (139/147 (95%) physicians in the intervention arm confirmed viewing an educational webcast about the 2.8MM probe-CMA. No physicians in the control arm received any specific educational material.

Recruitment of pediatrician participants was carried out by a mail campaign directed at a random subset of nationally representative lists of 25,000 general and 5,000 sub-specialty practice physicians. Recruit ran from August to September 2014, and letters were sent to 1,000 physicians randomly selected from the lists, explaining the purpose of the study and inviting physicians to participate. Eligibility for participation in the study was assessed using a physician questionnaire. Physicians had to be (1) either board-certified pediatric neurologists, developmental pediatricians or general pediatricians, (2) English-speaking, (3) practicing in a community/non-academic based setting, (4) accessible by Internet, and (5) have no prior experience with the 2.8MM probe-CMA test. Additionally, physicians who met the initial eligibility requirements were required to complete both a physician survey and two rounds of cases to remain in the study. In total, 232 respondents fulfilled the eligibility criteria and consented to participate (Fig 1). These physicians were randomized into control and intervention groups, using a 1:2 control to intervention ratio. Assignment into the arms was based on simple random allocation generated from Microsoft Excel’s random function. Between Rounds 1 and 2 of data collection, physicians in the intervention arm received a 25-minute webinar on the 2.8MM probe-CMA test and were provided with the 2.8MM probe-CMA test results specific to their patient if they chose to order and/or view the results in Round 2.

### Clinical Performance and Value® Vignettes

Clinical utility was measured at baseline (Round 1) and again after four weeks (Round 2) using CPV® vignettes for both groups. These vignettes are a widely-used, validated means for assessing differences in clinical practice and inherent variation in care, independent of case [14, 18–20]. In each vignette, the general and sub-specialist pediatricians were asked to provide open-ended responses regarding the clinical care they would provide for the simulated patient. Responses were scored in 5 domains: taking a medical history, performing a physical examination, ordering appropriate laboratory and imaging tests, assessing disease activity and physical function, and prescribing treatment. Explicit quality of care scoring criteria were established prior to study administration and were derived from the peer-reviewed medical literature and expert opinion by a physician board certified in Medical Genetics and Pediatrics in conjunction with a panel of clinical physicians. All participant responses were scored against these explicit scoring criteria by clinical physicians blinded to the participant’s identity. Overall CPV scores and scores with each domain represent the percentage of physician answers that addressed each of these evidence-based criteria, and range from 0 to 100%. CPVs have been linked to outcomes data in other studies, wherein a 3–5% increase in scores is associated with an improvement in outcomes and is the threshold used for clinical significance [21].

### Cases

The vignettes used in this study simulated clinical encounters involving simulated pediatric patients presenting with an atypical phenotype and/or developmental or intellectual deficits. Patient vignettes in this analysis were developed around five clinical syndromes describing a range of abnormal phenotypes with underlying genetic diagnoses causal of developmental or intellectual delay. Attention was given to conditions in which an accurate diagnosis would lead to change in treatment.

These cases were used to evaluate the impact of CMA testing and the 2.8MM probe-CMA testing on patient diagnosis and management. The order of the cases was randomly assigned. To assess physician, patient and practice characteristics, as well as physicians’ familiarity and experience with genetic testing, we also administered a questionnaire to all physicians prior to baseline.

All participants in both rounds could order and receive results from existing CMA tests; provision of the 2.8MM probe-CMA test results was restricted to Round 2 intervention group physicians. CMA testing options available to the physicians included 180K array, high resolution CMA, and the 2.8MM probe-CMA test. The five cases comprised three groups for CMA testing results: the first category of cases yield similar results regardless of the CMA selected; the second yields normal (i.e., no abnormality) or incomplete results for the 180K test, but abnormal results (i.e., genetic findings) using high resolution CMA and the 2.8MM probe-CMA test; and the third group yields normal 180K and high resolution CMA results but the 2.8MM probe-CMA test detects the abnormality (Table 1).

**Table 1:** Detail of CPV Vignettes and Grouping.

Diagnoses	Group
Hunter Syndrome, with a ruptured right tympanic membrane	1. Any CMA, including the 2.8MM probe-CMA, detects the genetic abnormality
Mosaic Turner syndrome with XY cell line, with risk for gonadoblastoma	2. 180K array normal*; high resolution CMA and the 2.8MM probe-CMA test detect the genetic abnormality
*SCN1A*-related seizure disorder or Dravet syndrome, uncontrolled myoclonic seizures	
Guanidinoacetate methyltransferase (GAMT) deficiency, failure to thrive	3. Other CMA tests report no abnormality but the 2.8MM probe-CMA test detects the genetic abnormality
FOXG1- related disorder or congenital Rett syndrome, global developmental delay/regression	

*For mosaic Turner syndrome, a low-density array for assessment of patient DNA collected via standard blood draw reports monosomy X [22].

### The 2.8MM Probe-CMA Testing and Reporting Process

The 2.8MM probe-CMA test is a whole-genome CMA that is optimized for clinical use in the setting of DD/ID and ASD in part through the addition of an additional 88,435 probes in genes/regions documented in the clinical literature to be associated or causative of these conditions [23]. Compared to other CMA testing platforms, it has a two-fold increase in detection rate for variants that underlie ASD (5–7% on typical CMA platforms to 12–14% on the 2.8MM probe-CMA test), and an overall increase in detection rate from 15–20% to over 30% for all suspected DD/ID cases including ASD [17,24]. The 2.8MM probe-CMA test results are provided in a detailed report that includes both physician-specific and parent-specific sections with a clear description of the underlying abnormality, suggestions for medical management and/or further testing, and recurrence risk. The increased detection rate and resulting clinical management recommendations from Lineagen’s 2.8MM probe-CMA test should increase the clinical utility of CMA testing when this format is used.

### Intervention

Before Round 2 data collection, subjects in the intervention arm were given an educational component describing CMA testing in general and the 2.8MM probe-CMA test specifically. This was a 25-minute web-based pre-recorded video, which participants watched at their convenience and provided an overview of genetic testing for ID/DD and described the 2.8MM probe-CMA test. Nearly all (138 of 149 intervention arm participants) confirmed viewing the intervention video. After viewing the short video, the intervention group completed three additional (randomly) administered cases and now had the opportunity to order and review the 2.8MM probe-CMA test results if they chose to do so. Physicians in the control arm also completed a second round of cases, but did not receive any information about the 2.8MM probe-CMA test and could not order or receive the 2.8MM probe-CMA test results.

### Analysis

First, we compared the intervention and control groups, and summary statistics were assessed for baseline provider and practice characteristics. For continuous and interval data, we used an independent samples t-test to determine the p-value. For categorical data, Fisher’s exact test was used to compare the distribution of the two groups. We then tested the main hypothesis: Does CMA testing in general and/or the 2.8MM probe-CMA testing specifically improve clinical utility? We measured changes in clinical utility using quality of care scores from the CPV® vignettes (e.g., ordering the proper diagnostic work, making appropriate referrals), which quantify the percentage of pre-determined evidence-based care criteria which were appropriately addressed by the physician while caring for the virtual patient. We further hypothesized that the pediatricians who ordered CMA, including the 2.8MM probe-CMA test, were more likely to improve their diagnostic accuracy and deliver specific treatments that were appropriate for the patient’s underlying condition. To test this, we estimated the intervention’s effect by performing a logistic regression and modeling the marginal effects for the specific diagnosis or treatment option of interest.

We compared the change (differences) in the outcome scores between Rounds 1 and 2 for both the intervention and the control groups. Univariate regression models using physician and practice characteristics were performed (S1 Table). Our *a priori* assumption was that all physician and practice characteristics would be of interest in the final model, and thus, our decision was to include all these variables in the final model, regardless of significance in the univariate model. After including these variables, we added in the relevant CMA, round, and intervention variables. 2.8MM is the explanatory variable describing those who ordered the 2.8MM probe in Round 2, and CMA is an explanatory variable for those who ordered CMA testing (other than the 2.8MM probe-CMA test) in either round. The 2.8MM variable measures the utility of the 2.8MM probe-CMA testing after its introduction in Round 2. We also analyzed the interaction of ROUND x CMA, to measure the differential change in the utility from CMA testing in Round 2 versus Round 1 and a possible learning effect spillover from the 2.8MM probe-CMA test to all CMA testing. The interaction terms between the 2.8MM probe-CMA test x Group 2 and the 2.8MM probe-CMA test x Group 3 tells us the differential effects on cases types of the 2.8MM probe-CMA test compared to any other CMA tests.

$$\begin{array}{l} Y_{jt}=\text{Vignette score at time}\;t,\;\text{physician}\;j\\ Y_{jt}∼Normal(\mu_{jt},\sigma_{e}^{2})\\ \mu_{jt}=\beta_{0}+\beta_{1}\text{CMA}+\beta_{2}(2.8MM)+\beta_{3}\text{ROUND}\times \text{CMA}+\text{control variables} \end{array}$$

## Results

### Provider Characteristics

After randomization, there were 147 participants in the intervention arm and 85 in the control arm. Ultimately, 202 physicians completed both rounds of data collection from September 2014 through January 2015, the predefined study period. 30 participants were dropped from the study (22 from the controls and 8 from the intervention arm) because they were determined to have not met initial inclusion criteria (n = 5), asked to be withdrawn from the study (n = 3), or did not complete the additional study requirements (n = 22) (Fig 1). There was no significant difference between the control and intervention groups by provider characteristic (Table *2*). Overall, the majority of the pediatricians were part of a hospital/health care system (61%). Over 90% were employed by their practice. 59% were pediatric specialists and 41% were general pediatricians.

**Table 2:** Characteristics of Physicians.

N = 202	Control = 63	Intervention = 139	p-value
**Female, %**	60.3%	57.6%	0.76
**Mean age, years (SD)**	45.4 (10.3)	46.4 (26.2)	0.76
**Specialty, %**			
Developmental and Other Pediatrician	30.2%	36.7%	0.19
Pediatric Neurologist	32.3%	20.9%	
General Pediatrician	36.5%	42.5%	
**Practice Ownership, %**			
Physician/Physician group	30.5%	22.3%	0.44
Hospital	59.3%	61.9%	
Community Health Center	6.8%	7.2%	
Other	3.4%	8.6%	
**Employed by practice, % Yes**	93.6%	92.8%	1.00

Practice characteristics, particularly as they relate to genetic testing, were varied (Table *3*). On familiarity with genetic testing, the average score was 2.9 on a scale of 1 to 5 and 44% rated themselves a 3. However, 85% felt that a geneticist was readily accessible to them if they needed consultation. Their patients were almost evenly split between commercial and public insurance patients (mostly Medicaid) and their practices were active with more than 58% of respondents reporting seeing over 50 general pediatric patients per week, while 30% reported seeing more than 20 patients with developmental disabilities per week.

**Table 3:** Practice Characteristics.

N = 202	Control	Intervention	p-value
**Familiarity with genetic testing (1-Very low to 5-Very high)**			
1–2	19.4%	35.5%	0.03
3	45.1%	42.8%	
4–5	35.5%	21.7%	
**Frequency of ordering genetic testing for patients with atypical phenotypes (1-Never to 5-Very frequently)**			
Average score	3.3 (1.4)	3.0 (1.3)	0.17
**Readily available genetics expert, %**			
Medical geneticist	35.6%	38.8%	0.52
Genetic counselor	6.8%	5.0%	
Both medical geneticist and genetic counselor	37.3%	43.2%	
Neither	20.3%	12.8%	
**Pediatric patients seen per week, %**			
<50	45.6%	40.6%	0.71
51–100	40.3%	46.4%	
>100	14.5%	13.0%	
**Proportion of all patients covered by, % (SD)**			
Medicare	8.0% (20.5%)	7.1% (16.3%)	0.74
Commercial	44.0% (26.9%)	45.0% (26.7%)	0.82
Medicaid	38.6% (29.0%)	41.0% (30.0%)	0.61
Self-pay and Other	10.5% (17.3%)	6.8% (13.0%)	0.10

### Ordering CMA Diagnostic Testing

Overall 36% of all physicians ordered CMA testing of any kind in Round 1 of the study and 44% ordered CMA testing in Round 2. Not surprisingly, almost no physicians in any of the study arms ordered the 2.8MM probe-CMA test in Round 1 (n = 5 or 1.1% of the sample) a pattern which persisted in the control arm in Round 2, where only 1 physician ordered the 2.8MM probe-CMA test. If the 2.8MM probe-CMA test was ordered in Round 1 or by a control arm physician in Round 2, the 2.8MM probe-CMA test results were delivered. Almost all of the increase in CMA testing in Round 2 came from physicians in the intervention group ordering specifically the 2.8MM probe-CMA test, where 20% (n = 60) of physicians ordered the test (Table 4).

**Table 4:** Proportion of Cases in Which Physician Ordered Genetic Testing by Round.

	Ordered any CMA testing of any kind, excluding the 2.8MM probe-CMA test, N (%)	Only Ordered the 2.8MM probe-CMA test, N (%)
**Round 1**	154 (35.2%)	5 (1.1%)
**Round 2**	168 (38.6%)	61 (14.0%)
**Round 1 Control**	61 (43.0%)	1 (0.7%)
**Round 2 Control**	57 (40.7%)	1 (0.7%)
**Round 1 Intervention**	93 (31.5%)	4 (1.4%)
**Round 2 Intervention**	111 (37.6%)	60 (20.3%)

### Link between Diagnostic Testing and CPV® Vignette Scores

CMA testing was associated with improved medical management, taken here as a surrogate for clinical utility. Those that ordered CMA testing had overall CPV quality scores 5.4% higher (p<0.001) than those that did not, indicative of clinically significant improvements in medical management (Table *5*). This difference did not change, in either magnitude or significance, in the second round of testing. However, the 2.8MM probe-CMA testing, which was only available in Round 2, resulted in CPV quality scores 7.2% higher than cases in which CMA was not ordered. This represents an additional 1.8% improvement in quality of care over cases in which other CMA tests were ordered, which is statistically significant (p<0.001).

**Table 5:** Multivariate linear regression model linking CMA and Utility: Overall CPV Scores.

	Overall CPV Score	
	Coefficient	p-value
CMA	5.43	<0.001
2.8MM probe-CMA	7.20	<0.001
Round Interacted with CMA	-0.79	0.678
Age (>40 years)	0.46	0.644
Gender (Female)	2.85	0.003
Generalist	3.24	0.008
Have a readily available genetics expert	1.43	0.214
Understanding of genetic testing as it relates to children with developmental disabilities (4–5)	-0.32	0.800
Frequency of ordering genetic tests (>3)	0.50	0.674
Employed	1.21	0.525
Practice size (>10)	-1.32	0.180
Practice ownership (physician group vs. other)	-3.58	0.003
Average days worked per week (>4)	0.15	0.890
Pediatric patients seen per week (>50)	0.04	0.970
Payer, Medicare/Medicaid >50%	0.50	0.611
Pediatric patients with DD, ID, ASD per week (<20)	0.01	0.990
Clinical Grouping of CPV Cases*		
Group 2 vs. Group 1	10.93	<0.001
Group 3 vs. Group 1	8.65	<0.001
Constant	34.17	<0.001

* CPV Group 1: Any CMA result and the 2.8MM probe-CMA test identify diagnosis; CPV Group 2: 180K array normal; high resolution CMA and the 2.8MM probe-CMA test identify diagnosis; CPV Group 3: Any standardly available CMA normal; the 2.8MM probe-CMA test detects abnormality. See Table 1 for more detail.

We looked at the three clinical groups detailed in Table 1 to determine if this improvement was from the additional clinical information provided by the 2.8MM probe-CMA test. By design, the 2.8MM probe-CMA test would provide similar diagnostic information as other CMA tests in Group 1, a more specific diagnosis compared to the 180k test in Group 2, and a more specific diagnosis compared to both the 180k and high resolution CMA in Group 3. A regression model including the interaction of the 2.8MM probe-CMA test with Groups 2 and 3 confirmed that overall CPV scores increased by 10.9% in Group 2 and 8.7% in Group 3 CPVs, compared to Group 1 (p = <0.001 for each Group). Two additional covariates were also associated with higher scores: generalist physicians (p = 0.008) and female physicians (p = 0.003).

For the five cases where the 2.8MM probe-CMA test offered a specific diagnosis, this was also associated with a statistically significant, 10.9% greater accuracy (p<0.001) for combined diagnosis and treatment scores (Table 6). This difference accounted for the majority of overall improvement in clinical utility noted in Table 5. Conversely, other CMA testing (defined as 180K, regular or high resolution CMA, but excluding the 2.8MM probe-CMA test) was not linked to significantly higher scores in the combined diagnosis and treatment domain, showing an increase of only 2.7% in the diagnosis and treatment scores (p = 0.122). In this analysis, neither covariate of generalist physician or gender of physician was associated with a significant increase in the combined score. However, those physicians who did not own their practice were more likely to accurately treat and diagnose their patients (p = 0.001). The effect of using the 2.8MM probe-CMA test was strong and positive in both Group 2 and Group 3.

**Table 6:** Multivariate linear regression model linking CMA and Utility: CPV Diagnosis and Treatment Scores.

	Diagnosis and Treatment Score	
	Coefficient	p-value
CMA	2.69	0.122
2.8MM probe-CMA	10.90	<0.001
Round Interacted with CMA only	-0.36	0.881
Age (>40 years)	-1.34	0.295
Gender (Female)	0.70	0.567
Generalist	2.37	0.129
Have a readily available genetics expert	2.81	0.059
Understanding of genetic testing as it relates to children with developmental disabilities (4–5)	1.79	0.268
Frequency of ordering genetic tests (>3)	0.16	0.915
Employed	0.47	0.848
Practice size (>10)	-0.92	0.468
Practice ownership (physician group vs. other)	-5.03	0.001
Average days worked per week (>4)	0.58	0.685
Pediatric patients seen per week (>50)	-0.39	0.781
Payer, Medicare/Medicaid >50%	0.11	0.928
Pediatric patients with DD, ID, ASD per week (<20)	-0.35	0.810
CPV Clinical Grouping*		
Group 2 vs. Group 1	10.91	<0.001
Group 3 vs. Group 1	2.65	0.093
Constant	16.49	<0.001

*CPV Group 1: Any CMA result and the 2.8MM probe-CMA test identify diagnosis; CPV Group 2: 180K array normal; high resolution CMA and the 2.8MM probe-CMA test identify diagnosis; CPV Group 3: Any standardly available CMA normal; the 2.8MM probe-CMA test detects abnormality.

Finally, we completed a secondary analysis, conducting a case-by-case evaluation of diagnostic and treatment practice changes associated with more accurate the 2.8MM probe-CMA test diagnosis (Table 7). Despite the lack of statistical significance, the disaggregated data were directionally suggestive of several benefits. For two out of the five conditions (Hunter syndrome and mosaic Turner syndrome), the 2.8MM probe-CMA test led to a statistically higher rate of accurate diagnosis, as seen in the improved diagnosis scores in the intervention group. Correct secondary diagnoses, such as gross motor delay for Hunter syndrome and uncontrolled myoclonic seizures for Dravet syndrome, were also more likely to be made for four of the five conditions (Table 7). For almost every condition studied there were some statistically significant differences in treatment. Examples of these treatment differences include: evaluation and therapy for gross motor delays (Hunter syndrome); anticipatory guidance and screening for heart disease (Turner syndrome); recommending early cognitive intervention (FOXG1-related disorder); referral to child neurologist and commencement of neuroleptics (Dravet syndrome). Many other treatments were directionally positive but not statistically significant and thus not causally established. However, in the Turner syndrome case, one important treatment that showed there was no difference between the intervention and control groups in either ordering or discussing the performing a prophylactic gonadectomy, despite the risk of a gonadoblastoma in these patients.

**Table 7:** Case specific diagnostic and treatment practice changes among the intervention group.

Case	Improvement in Intervention over Controls, %*	P-Value
**Hunter syndrome–diagnosis (n = 145)**	45.4	**<0.01**
Secondary diagnoses/problem list: Ruptured tympanic membrane	7.0	0.62
Secondary diagnoses/problem list: Gross motor delay	8.3	**0.04**
Enzyme replacement therapy (ERT) with idursulfase	7.1	0.65
Physiotherapy, occupational therapy, speech therapy	10.2	0.53
Continued ENT evaluation for hearing	15.7	0.43
**Guanidinoacetate methyltransferase (GAMT) deficiency–diagnosis (n = 152)**	4.7	0.27
Secondary diagnosis/problem list: Failure to thrive / aspiration feeding difficulties	7.08	0.67
Creatinine monohydrate supplement 300-100mg/kg/day in 2 divided doses	0.1	0.78
Low arginine diet/ high ornithine diet	6.3	1.00
**Mosaic Turner syndrome with XY cell line–diagnosis (n = 140)**	26.1	**<0.01**
Continued evaluation of developmental milestones	25.3	**0.06**
Advice regarding complications from bicuspid aortic valve	23.8	0.12
Screening 2D-echo for valve abnormalities	13.4	**0.05**
Growth hormone administration and Estrogen replacement therapy at 12–15 years of age	9.96	0.48
**FOXG1-related disorder or congenital Rett syndrome–diagnosis (n = 151)**	11.2	1.00
Therapy that promotes ambulation, balance, and hand use	5.1	**0.06**
Monitoring for seizure activity	14.9	0.76
Regular monitoring for scoliosis	1.7	1.00
Early cognitive intervention	27.5	**0.03**
**SCN1A-related seizure disorder or Dravet syndrome–diagnosis (n = 142)**	9.8	0.88
Secondary diagnoses/problem list: Uncontrolled myoclonic seizures	9.5	0.75
Advise family about avoiding seizure triggers (rapid changes in environmental and/or body temperature, illness, stress, overexcitement, patterns, and flickering lights) whenever possible	0.8	0.94
Referral to child neurologist	18.2	**<0.01**
Adaptive equipment—Video monitoring, protective helmets, cooling vests, pulse oximeters, seizure alarms, and glasses with colored lenses (for photosensitivity)	14.2	0.35

*The modeled round-over-round differential improvement of intervention compared to controls. Calculated as: (% Intervention correct in round 2 - % Intervention correct in round 1)—(% Control correct in round 2 - % Control correct in round 1).

## Discussion

Appropriate assessment of new health care interventions, particularly diagnostic technologies, requires high quality information on the clinical utility, economic value, affordability, and population health benefits of the technology in question [25]. As a reflection of this, assessing clinical utility—demonstrating the usefulness of a test in clinical practice—is now a significant hurdle required by payors before offering insurance coverage for most new technologies [26]. As government and commercial payors struggle to make decisions regarding reimbursement, demonstrating that additional clinical information is useful and leads to changes in what a provider does is critically important for any vulnerable population [27].

Developmental delays, including autism spectrum disorder, have both a common phenotypic expression and are a reflection of myriad different genetic conditions, each with its own clinical characteristics that require unique therapeutic interventions. Assessing these patients clinically, however, is time consuming and difficult, leading to missed diagnoses for thousands of children every year [28,29]. To overcome this, professional societies recommend that genetic testing, specifically CMA, be done as first tier diagnostic evaluation of these conditions [7–10].

We sought to clarify the evidence around the utility of CMA testing for patients with developmental delay associated with five specific genetic copy number variants (CNVs) detected by CMA testing. We did this by using an innovative measurement tool, CPVs, which controls for patient case mix, overcomes the problem of collecting an adequate number of patients with a specific (rare) genetic disorder and allows us to conduct a randomized controlled experiment of related physician behavior and patient management. CPVs are a validated measure of clinical practice that has been widely used to serially measure clinical performance [20,30]. The tool has proven particularly valuable in clinical utility studies determining whether new technologies change clinical decision making and associated spending [18]. Using this tool, we examined whether CMA testing in general, and the 2.8MM probe-CMA test testing specifically, changed provider practice for five different patient genotypes.

We found that CMA testing did indeed improve the quality of care and clinical decision making among more than 200 general and sub-specialty pediatricians. When physicians used the 2.8MM probe-CMA test, an optimized whole-genome high resolution CMA, we found an increased causal relationship between testing and improved clinical utility. Physician users of the 2.8MM probe-CMA test had a statistically and clinically significant increase of 7.2% in their overall utility scores over those physicians who use no CMA testing at all and a 1.8% marginally higher score over those who use standard CMA testing. The effect of the 2.8MM probe-CMA test is even more notable on diagnosis and treatment scores, arguably the most critical part of the care process. Scores of the 2.8MM probe-CMA test users for the combined domain score for diagnosis and treatment were 10.9 percentage points higher than those who used no CMA testing at all. The 2.8MM probe-CMA test was particularly useful in individuals who harbor a pathogenic CNV in regions covered by the custom probes contained on that CMA to optimize clinical interpretation in cases of DD, ASD and ID, including but not limited to the genes for GAMT deficiency (GAMT) and congenital Rett syndrome (FOXG1-related disorder). Most of the commercially available CMA platforms cannot detect these, and other genetic abnormalities targeted by the 2.8MM probe-CMA test. The increased clinical utility of the 2.8MM probe-CMA test also extends to individuals affected by syndromes such as Mosaic Turner syndrome with an XY cell line and Dravet syndrome, which can be diagnosed by some other CMA tests available on the market. We hypothesize that this increase in clinical utility is driven by the comprehensiveness and clarity of the 2.8MM probe-CMA test report, which includes specific treatment recommendations.

We observed opportunities to improve the quality of care delivered to all patients with DD/ID and ASD. At baseline, only 37% of physicians ordered any CMA for their patients. Following the addition of the 2.8MM probe-CMA test, after providing a simple intervention, CMA testing increased significantly to 44%. CMA testing at baseline was surprisingly low suggesting that professional medical guidelines are either not widely known or are not used.

By analyzing the specific aspects of care provided to these patients, we found that the 2.8MM probe-CMA test provided benefits in many areas such as identifying the primary diagnosis of the DD/ID syndrome, managing secondary diagnoses, and defining treatment. The treatment improvements led to instances of proper treatment and presumably earlier therapy. Indeed, successful management of ID/DD/ASD is predicated on recognition of any underlying syndrome. Other studies will be needed to determine if earlier, more precise, diagnosis from CMA testing leads to less unnecessary testing or less testing overall.

This study has several limitations. The control group received neither orientation on genetic testing nor the 2.8MM probe-CMA test results, thus it is difficult to tease apart the effect from the 2.8MM probe-CMA test results from that of the educational intervention on genetic disorders and testing. Second, the five diseases that were the focus of the CPVs are each relatively rare like most DD, ASD and ID syndromes, thus results here may not necessarily be extrapolated to all underlying syndromes. We do suspect however that regardless of the syndrome, the challenges of arriving at a genetic diagnosis and developing an appropriate therapeutic plan for the individual are common across a number of disease types. Thirdly, this study did not address issues related to variants of unknown significance, additional secondary possibly actionable variants, or point mutations necessitating genomic sequencing approaches to detect. As genetic tests become increasingly detailed and complex, these issues will benefit from future follow-up work. Finally, this analysis focused on CMA testing, which in general will detect cases of these conditions due to larger deletions and duplications, not point mutations. Future studies of this type will be needed to address the contribution of point mutations by emerging next-generation sequencing technology.

Studies to determine the clinical utility and impact of diagnostics and interventions for rare diseases are particularly challenged by the difficulty in locating and identifying an adequate number of patients for study. Those who attempt to undertake assessments of clinical utility using patient level data are additionally challenged by difficulty controlling for case mix which is a routine issue in any patient level study. This challenge is further compounded when dealing with rare diseases and small numbers of study participants. The CPV vignettes provide a standardized method to analyze how physicians with different backgrounds and from various settings assess and treat the same patient. While the CPV approach does not use actual patient level data, they uniquely provide a standardized method to analyze how physicians with different backgrounds and from various settings would assess and treat the same patient. Our results show that CMA in general and specifically the 2.8MM probe-CMA test provides improved medical management in children with developmental delay, and therefore meet criteria for clinical utility.

## Supporting Information

S1 Table Univariate regression model linking CMA and Utility: Overall CPV Scores. (DOCX)

S1 Checklist CONSORT 2010 checklist of information to include when reporting a randomized trial. (DOC)

S1 Protocol Clinical study protocol. (PDF)

S1 File Protocol approval with modifications. (PDF)
