# Supplementary material for: Clinical Utility of a Comprehensive, Whole Genome CMA Testing Platform in Pediatrics: A Prospective Randomized Controlled Trial of Simulated Patients in Physician Practices
Source: PLoS One. 2016 Dec 30;11(12):e0169064. doi: 10.1371/journal.pone.0169064 (PMC5201278; doi:10.1371/journal.pone.0169064)
Supplement: S1 Table — (DOCX) [file pone.0169064.s001.docx]

**S1 Table. Univariate regression model linking CMA and Utility: Overall CPV Scores**

|  | **Overall CPV Score** | |
| --- | --- | --- |
|  | Coefficient | p-value |
| CMA | 4.16 | <0.001 |
| 2.8MM probe-CMA | 6.28 | 0.001 |
| Round | 1.14 | 0.227 |
| Age (>40 years) | 0.35 | 0.728 |
| Gender (Female) | 2.89 | 0.003 |
| Generalist | 1.79 | 0.072 |
| Have a readily available genetics expert | 2.58 | 0.031 |
| Understanding of genetic testing as it relates to children with developmental disabilities (4-5) | -0.54 | 0.629 |
| Frequency of ordering genetic tests (>3) | -0.29 | 0.761 |
| Employed | 3.09 | 0.109 |
| Practice size ( >10) | -0.81 | 0.417 |
| Practice ownership (physician group vs. other) | -3.32 | 0.003 |
| Average days worked per week (>4) | -0.61 | 0.555 |
| Pediatric patients seen per week (>50) | -0.69 | 0.485 |
| Payer, Medicare/Medicaid >50% | 2.26 | 0.021 |
| Pediatric patients with DD, ID, ASD per week (<20) | 0.54 | 0.599 |
| Clinical Grouping of CPV Cases* |  |  |
| Group 2 vs. Group 1 | 10.72 | <0.001 |
| Group 3 vs. Group 1 | 8.79 | <0.001 |
