# Supplementary material for: Clinical Utility of a Comprehensive, Whole Genome CMA Testing Platform in Pediatrics: A Prospective Randomized Controlled Trial of Simulated Patients in Physician Practices
Source: PLoS One. 2016 Dec 30;11(12):e0169064. doi: 10.1371/journal.pone.0169064 (PMC5201278; doi:10.1371/journal.pone.0169064)
Supplement: S1 Protocol — (PDF) [file pone.0169064.s003.pdf]

CLINICAL STUDY PROTOCOL

**Protocol Title:**	Establishing the Clinical Utility (ECU) of First Step[Dx] PLUS and NextStep[Dx] PLUS Study
**Protocol Number:**	01-LIN-2014
**Study Sponsor:**	Lineagen, Inc.<br>2677 Parleys Way<br>Salt Lake City, UT 84109<br>Phone +1 (801) 931-6200<br>Fax +1 (801) 931-6201
**Protocol Date:**	11 June 2014

CONFIDENTIALITY STATEMENT

This document contains confidential information. By accepting this document, you agree to maintain the information as confidential and to use it only for the purpose of conducting the study.

PROTOCOL ACCEPTANCE PAGE

Protocol Title: Establishing the Clinical Utility (ECU) of First Step^{Dx} PLUS and NextStep^{Dx} PLUS Study

Protocol Number: 01-LIN-2014

Protocol Date: 11 June 2014

Approvals:

John W Peabody MD, PhD, FACP<br>Principal Investigator for the PMCS<br>President, QURE, LLC	6/12/14<br>Date
Michael Paul, PhD<br>CEO, Lineagen	06/12/14<br>Date
Rena Vanzo, MS, LCGC<br>Director, Clinical Genetic Services	06/12/14<br>Date

TABLE OF CONTENTS

REVISION HISTORY 4
ACRONYMS 5
STUDY SYNOPSIS 6
1. BACKGROUND INFORMATION AND RATIONALE 9
2. STUDY DESIGN 10
3. STUDY OBJECTIVES 12
4. ELIGIBILITY CRITERIA 12
5. TREATMENT OF SUBJECTS 13
6. STUDY PROCEDURES 14
7. STUDY DATA COLLECTION 14
8. STATISTICAL ANALYSIS 14
9. ADMINISTRATIVE CONSIDERATIONS 17
REFERENCES 19

REVISION HISTORY

Revision	Originator	Date Effective	Nature of Change
A	L DeMaria	4 June 2014	Initial Release
B	L DeMaria	10 June 2014	Incorporation of Rena Vanzo / Lineagen edits
C	L DeMaria	11 June 2014	Incorporation of M Paul edits

Confidential Proprietary Property of QURE Healthcare, LLC and Lineagen, Inc.

ACRONYMS

ASD	Autism spectrum disorder
CMA	Chromosomal microarray
CPV®	Clinical Performance and Value
DD	Developmental delay/disability
ID	Intellectual disability
SD	Standard deviation

STUDY SYNOPSIS

Protocol Title Establishing the Clinical Utility (ECU) of First Step^Dx PLUS and NextStep^Dx PLUS Study

Background

The American Academies of Pediatrics, Neurology and Child and Adolescent Psychiatry consider the genetics evaluation part of the comprehensive assessment of individuals with disorders of postnatal development including developmental delay (DD) autism spectrum disorder (ASD), and intellectual disability (ID). Due to the added clinical detection rate of chromosomal microarray analysis (CMA) over basic genetic testing technologies (such as standard karyotyping), in 2010, the American College of Medical Genetics recommended CMA as a first-tier diagnostic genetic evaluation. Lineagen's diagnostic genetic test, FirstStep^Dx PLUS, includes the **most comprehensive, whole-genome CMA** currently available for clinical use. It has a two-fold increase in detection rate for variants that underlie ASD (from 5-7% on typical CMA platforms to 12-14% on FirstStep^Dx PLUS; Matsunami et al. 2013, Matsunami, et al 2014) and an overall increase in detection rate (from 15- 20% to over 30%). Once a genetic diagnosis is obtained, screening and management of associated life-threatening medical risks (seizures, tumors, renal disease, heart disease), administration of indicated medications and therapies, and avoidance of contraindicated treatments can be implemented (Coulter and Irons and Ellison references).

Appropriate assessment of new health care interventions or technologies requires information on the clinical utility, economic value, affordability, and public health benefits of these interventions (Phillips et al. 2014; Peabody et al. forthcoming). Clinical utility—demonstrating the usefulness of a test for clinical practice—is now the most significant hurdle facing diagnostic technology companies. Today, without scientific evidence of clinical utility, even the most promising technologies may not be covered and reimbursed by payors.

Study Objective

To determine if use of FirstStep^Dx PLUS and Next Step^Dx PLUS are associated with higher clinical quality, less variability in clinical practice, and lower costs from decreased resource utilization.

Study Design

The study uses a randomized controlled study design of pediatric neurologists and developmental pediatricians and front-line (primary care) pediatricians. Physicians will be randomly assigned to a control or one of two intervention arms.

- Pre-intervention/baseline assessment: will be done using pediatric CPV® vignettes and a physician questionnaire to <u>both</u> control and intervention arms.
- Intervention: 2-4 weeks after baseline introduce FirstStep^Dx PLUS and NextStep^Dx PLUS to the intervention group of doctors using a webinar format to describe the application and utilization of the two assays.

Confidential Proprietary Property of QURE Healthcare, LLC and Lineagen, Inc.

Post-intervention: 4 weeks post-intervention, a second round of pediatric CPV® vignettes will be administered to both control and intervention arms. FirstStep^{Dx} PLUS results will be available to order as part of the vignettes and results will be presented as a downloadable PDF for intervention group 1; FirstStep^{Dx} PLUS and NextStep^{Dx} PLUS will be available for physicians in intervention group 2.

Study instruments

The Clinical Performance and Value Vignettes (CPV®) used in this study simulate a clinical encounter for individuals with an atypical phenotype and clinical presentation indicative of a possible genetic disorder. Each physician will respond to open-ended questions regarding the clinical care they would provide for that patient. These responses are scored in five domains (taking a medical history, conducting a physical examination, ordering appropriate tests, making a diagnosis, and prescribing treatment and therapies against explicit evidence and criteria as determined by the literature and medical associations. Results are presented as percentage correct. Each case will take approximately 15 minutes to complete. All case responses will be completed online and kept confidential.

A questionnaire will also be administered to all physicians at the beginning of the first round of CPV vignette data collection. This questionnaire will assess physician and practice characteristics.

Outcome Measures

Primary Outcome

- Difference in combined diagnostic and treatment CPV® domain score post-intervention versus baseline comparing intervention and control groups

Secondary Outcomes

- Difference in utilization of appropriate treatment including indicated versus unnecessary therapy pre- and post-intervention between intervention and control groups
- Difference in utilization of laboratory testing such as fluorescence *in situ* hybridization (FISH) analyses, global assessment, and utilization of other laboratory tests pre- and post-intervention, between intervention and control groups
- Difference in overall Clinical Performance and Value Vignettes (CPV®) scores post-intervention versus baseline between intervention and control groups

Study Population

225 specialists and general pediatricians (specialists include pediatric neurologists and developmental pediatricians) will be recruited and invited to participate in the study. 70% of the sample will be comprised of specialist physicians and 30% of primary care pediatricians.
Inclusion eligibility criteria cover:

- A minimum of 2 years post residency, no more than 30 years in practice
- Board-certified
- No prior use of either the FirstStep^{Dx} PLUS or NextStep^{Dx} PLUS assays

- Minimum threshold of pediatric patients currently being treated: >30 patients

Physician Recruitment

Providers will be contacted by recruitment letter sent via USPS and then by email and/or telephone. A dedicated script will be used to assess eligibility and willingness to participate. Physicians will be randomly selected from lists of approximately 5,000 specialists and 25,000 general pediatricians. Physician privacy and confidentiality will be maintained in accordance with HIPAA regulations.

Informed consent will be obtained by having physicians read the consent and provide their signature and date and time of consent. Once this is obtained, physicians will be considered enrolled into the study.

Sample Size

The study is adequately powered to detect differences in the combined diagnosis and treatment score as measured by the CPVs®. A sample size of 75 in each arm (225 total) provides over 80% power to detect an estimated 6-7% difference in scores with alpha=0.05. Due to possible attrition, extra subjects may be enrolled to ensure that at least 225 subjects will be used in the analysis.

Study Hypotheses

1) Clinical practice, specifically decisions around treatment of individuals with disorders postnatal development including DD, ASD, and ID will vary widely among all physician types. This documentation of variation in practice will demonstrate the need for a new diagnostic service that FirstStep^{Dx} PLUS and NextStep^{Dx} PLUS would fulfill
2) FirstStep^{Dx} PLUS and NextStep^{Dx} PLUS will improve the quality and appropriateness of care and therapeutic plans, as measured by the number of physicians that correctly act on the results of the assay

Data Analysis

Summary statistics will be determined for all variables. Simple cross tabulations will be prepared as part of the data resolution process. The calculation of basic descriptive statistics is used to check distributions and find outliers and out-of-range data. Numerical variables will be characterized in terms of means and standard deviations or by medians and interquartile range for skewed or highly non-normally distributed variables. For hypothesis testing, tests will be conducted at a two-sided $\alpha = 0.05$.

With experimental data we can estimate the effect of FirstStep^{Dx} PLUS or the sequential testing of FirstStep^{Dx} PLUS and NextStep^{Dx} PLUS on the primary outcomes comparing treatment and control groups. We will use a difference in difference analysis approach comparing differences between groups before and after intervention. We will control for all potential confounders, including physician-level characteristics, such as age, gender, specialty, years in practice, and practice-level characteristics—multi- versus single specialty practice, rural/urban location, and payor mix.

1. BACKGROUND INFORMATION AND RATIONALE

1.1 Background Information

Approximately 16% of U.S. individuals have a form of developmental disability (Boyle 2011). Genes are one of the few "clinically validated factors" known to be causal for DD, ASD, and ID. As an example, ASD has been found to be up to 70% genetic in origin (Ronald and Hoekstra 2011).

The American Academies of Pediatrics, Neurology, and Child and Adolescent Psychiatry consider the genetics evaluation part of the comprehensive assessment of individuals with disorders of postnatal development including autism spectrum disorder (ASD) (Shen et al 2010; Michelson et al 2011; Volkmar et al 2014). Due to the added clinical detection rate of chromosomal microarray analysis (CMA) over basic genetic testing technologies (such as standard karyotyping), in 2010, the American College of Medical Genetics recommended CMA as a first-tier diagnostic genetic evaluation (Manning and Hudgins 2010). Lineagen's diagnostic genetic testing, FirstStep^{Dx} PLUS, includes the **most comprehensive, whole-genome CMA** currently available for clinical use. It has a two-fold increase in detection rate for variants that underlie ASD (from 5-7% on typical CMA platforms to 12-14% on FirstStep^{Dx} PLUS; Matsunami, et al. 2013) and an overall increase in detection rate (from 15- 20% to over 30%). The increased detection rate and resulting clinical management changes of Lineagen's FirstStep^{Dx} PLUS warrants acceptance as *the* first-tier "standard of care" diagnostic genetic test for individuals with disorders of postnatal development. Any individual with a disorder of postnatal development, including ASD, who is receiving an alternate CMA platform is receiving a scientifically and clinically inferior CMA, with a lower established detection rate compared to FirstStep^{Dx} PLUS. In certain cases, when whole-genome CMAs are inconclusive, further diagnostic testing is warranted. Lineagen's NextStep^{Dx} PLUS sequencing-based test outpaces other sequencing-based tests to provide actionable clinical information to the pediatrician and pediatric specialist (Matsunami et al., 2014). The higher detection rate of both FirstStep^{Dx} PLUS and NextStep^{Dx} PLUS results in an increased ability to identify the underlying genetic diagnosis for the patient. Once a genetic diagnosis is obtained, screening and management of associated life-threatening medical risks (seizures, tumors, renal disease, heart disease), administration of indicated medications and therapies, and avoidance of contraindicated treatments can be implemented (Coulter et al. 2011; Ellison et al. 2012).

Appropriate assessment of new health care interventions or technologies requires information on the clinical utility, economic value, affordability, and public health benefits of these interventions (Phillips et al. 2014; Peabody et al. forthcoming). Clinical utility—demonstrating the usefulness of a test for clinical practice—is now the most significant hurdle facing diagnostic technology companies. Today, without scientific evidence of clinical utility, even the most promising technologies may not be covered and reimbursed by payors. Thus, early identification of a data analysis plan that generates evidence on clinical utility is essential for today's reimbursement environment and must provide scientifically rigorous estimates of how a test will change clinical practice outcomes for patients and populations.

Confidential Proprietary Property of QURE Healthcare, LLC and Lineagen, Inc.

1.2 Rationale for the Study

Appropriate assessment of new health care interventions or technologies requires information on the clinical utility, economic value, affordability, and public health benefits of these interventions (Phillips et al. 2014; Peabody et al. forthcoming). Clinical utility—demonstrating the usefulness of a test for clinical practice—is now the most significant hurdle facing diagnostic technology companies. Today, without scientific evidence of clinical utility, even the most promising technologies may not be covered and reimbursed by payors. Thus, early identification of a data analysis plan that generates evidence on clinical utility is essential for today's reimbursement environment and must provide scientifically rigorous estimates of how a test will change clinical practice outcomes for patients and populations.

2. STUDY DESIGN

The study is an interventional longitudinal study design of physician practice. 225 specialist and general pediatricians without previous exposure to FirstStep[Dx] PLUS or NextStep[Dx] PLUS will be selected from a nationally representative list of approximately 25,000 board-certified pediatricians and 5,000 specialists. These physicians will be **randomized** into one of three arms: to receive information regarding FirstStep[Dx] PLUS ("Intervention A"), to receive information about FirstStep[Dx] PLUS <u>and</u> NextStep[Dx] PLUS ("Intervention B") for use in clinical practice, and controls not receiving any intervention.

2.1 Intervention

The respective interventions will be administered to those randomized into one of the two intervention arms. The intervention consists of information regarding FirstStep[Dx] PLUS alone (Intervention Group A) or sequential testing of FirstStep[Dx] PLUS <u>and</u> NextStep[Dx] PLUS, (Intervention Group B) which will be offered through emailed materials, a pre-recorded 20-minute webinar, and access to questions of the Principal Investigator of the ECU Study and the Lineagen Genetic Counselor.

Group A: Randomized group of 75 physicians who are FirstStep[Dx] PLUS and NextStep[Dx] PLUS naïve at baseline and receive the intervention of FirstStep[Dx] PLUS alone.

Group B: Randomized group of 75 physicians who are FirstStep[Dx] PLUS and NextStep[Dx] PLUS naïve at baseline and receive the intervention of FirstStep[Dx] PLUS <u>and</u> NextStep[Dx] PLUS.

Group C: Randomized group of 75 physicians who are FirstStep[Dx] PLUS and NextStep[Dx] PLUS naïve at baseline and do not receive the intervention (controls).

2.2 Study Instruments

A Physician Questionnaire will be administered to all physicians. This questionnaire will assess physician, patient and practice characteristics. Data gathered from this set of questions will become part of the baseline (pre-intervention) assessment and used for analysis. At the baseline assessment, Clinical Performance and Value (CPVs) Vignettes, a validated tool to

Confidential Proprietary Property of QURE Healthcare, LLC and Lineagen, Inc.

measure physician performance and behavior will be used (Peabody et al 2004). CPV vignettes have been used to establish clinical utility in the molecular diagnostic space. (Peabody et al 2012)

The CPV® vignettes used in this study will simulate a clinical encounter for a patient with an atypical clinical presentation indicative of a possible genetic developmental disorder. Each physician will provide open-ended responses regarding clinical care. These responses are scored in five domains (taking a medical history, performing a physical examination, ordering appropriate tests, making a diagnosis and prescribing treatment against explicit evidence and criteria as determined by the literature and by expert physicians. Results are presented as percentage correct controlling for primacy effects using case within pair randomization. Each case will take approximately 15-20 minutes to complete. All case responses will be completed electronically online and confidential. No physician or practice names are used when reporting the results of the study.

Round 2 of data collection, or post-intervention assessment, will occur 4 weeks after the introduction of the intervention.

CPV scores will be used to compare baseline practice with practice post-intervention in the 3 different physician groups/arms.

This study will test the following hypotheses:

1. Clinical practice, specifically decisions around treatment of individuals with disorders of postnatal development including DD, ASD, and ID will vary widely among all physician types. This documentation of variation in practice will demonstrate the need for a new diagnostic service that FirstStep^{Dx} PLUS and NextStep^{Dx} PLUS would fulfill.

2. FirstStep^{Dx} PLUS and NextStep^{Dx} PLUS will improve the quality and appropriateness of care and therapeutic plans, as measured by the number of physicians that correctly act on the results of the assay.

   This hypothesis will be tested through total of 9 CPVs consisting of 3 CPVs cases for each of 3 case archetypes as follows.

   - Case A: Patients who are currently undiagnosed and for whom FirstStep^{Dx} PLUS or would initiate appropriate therapy
   - Case B: Patients who are wrongly—or incompletely—diagnosed and for whom FirstStep^{Dx} PLUS would change therapy (i.e., terminate inappropriate or unnecessary therapy and initiate appropriate therapy)
   - Case C: Patients who are undiagnosed by FirstStep^{Dx} PLUS testing and for whom NextStep^{Dx} PLUS testing would initiate appropriate therapy and/or earlier intervention will result in better outcomes

The CPVs™ will be randomly assigned to physicians for each to complete 3 CPVs™ (one from each type). Each of the CPVs™ will be scored by physicians for changes in clinical practice including treatment changes, frequency of follow up, laboratory tests and imaging studies ordered - see for scoring sheet.

3. STUDY OBJECTIVES

The objective of this study is to assess how FirstStep^{Dx} PLUS (and NextStep^{Dx} PLUS) testing affects the variability of clinical practice and resultant impact.

3.1 Primary Endpoint

Differences in CPV diagnosis/treatment domain score post-intervention versus baseline for the intervention and control group physicians).

3.2 Secondary Endpoints

- Difference in utilization of appropriate treatment including indicated versus unnecessary therapy pre- and post-intervention between intervention and control groups
- Difference in utilization of laboratory testing such as fluorescence *in situ* hybridization (FISH) analyses, global assessment, and utilization of other laboratory tests pre- and post-intervention, between intervention and control groups
- Difference in overall Clinical Performance and Value Vignettes (CPV®) scores post-intervention versus baseline between intervention and control groups

4. ELIGIBILITY CRITERIA

4.1 Description of subjects

Practicing physicians will be the study subjects with the following eligibility criteria.

4.1.1 Inclusion Criteria

Subjects must meet the following criteria to be enrolled in the ECU Study:

1. Provide consent to participate in the study
2. Currently practicing board-certified physician in the following specialty areas:
   a. Pediatric neurologists
   b. Developmental pediatricians
   c. General pediatricians
3. Have practiced as a board-certified physician for greater than 2 but less than 30 years.
4. English-speaking
5. Community / non-academic based practice setting
6. ≥30 pediatric patients under care annually
7. Access to the internet

4.1.2 Exclusion Criteria

Subjects will not be included in the ECU Study if they meet any of the following exclusion criteria:

1. Not board certified in their respective area of care
2. Academic-based practice
3. Have previously used FirstStep^{Dx} PLUS and/or NextStep^{Dx} PLUS in their care delivery
4. Have practiced as a board-certified physician for less than 2 or greater than 30 years.
5. Follow <30 pediatric patients annually
6. Non-English speaking
7. Unable to access the internet

4.2 Subject Screening and Enrollment

Physicians will be screened by trained recruiters for inclusion/exclusion criteria and willingness to participate in the protocol.

Informed consent will be obtained by having physicians read the consent and provide their signature and date and time of consent. Once this is obtained, physicians will be considered enrolled into the study.

4.3 Subject Withdrawal

If a participant fails to complete three CPV vignettes, they will be withdrawn from the study and replaced with another physician. Any CPVs from the withdrawn subject will be excluded from the analysis.

4.3.1 Withdrawal Criteria

Reasons for study withdrawal may include but are not limited to:

- Noncompliance with study procedures.
- Physician's right to withdraw consent at any time during the study with or without stated reason.

4.3.2 Documentation of Withdrawal of Subjects

The reason for withdrawal of any physician from the study will be appropriately documented.

5. TREATMENT OF SUBJECTS

The subjects are physicians. There is limited risk to participation. Risk would be loss of confidentiality and revelation of their score. Participants (physicians) will be informed of these risks and asked to provide IRB approved consent before participation. Participation is strictly voluntary. Subjects will be appropriately compensated for their participation in the study.

6. STUDY PROCEDURES

At enrollment, each eligible physician who agrees to participate will be assigned a unique subject identification number to be applied on all forms. Only the study team will have access to the subject's identity. The subject's name or other identifying information will not be used in analysis or reporting.

The main study procedures or assessments will include but are not limited to: collection of demographic and practice information during the screening process and completion of CPVs.

6.1 Pre-Screening and Eligibility

The evaluations for inclusion and exclusion criteria will include board certification, practice type and experience with FirstStep^{Dx} PLUS and/or NextStep^{Dx} PLUS.

6.2 Completion of CPVs™

Enrolled physicians will be provided a website address, user name and password to access the CPVs™. Each CPV™ is expected to take approximately 15-20 minutes. Participants will be given 7 days to complete the CPVs. If the CPVs are not completed within this time frame, the physician will be contacted and requested to complete the CPVs. Physicians will be reminded to complete the CPVs by phone calls and/or emails. If after 10 business days, they still have not completed the CPV, they will be withdrawn from the study.

7. STUDY DATA COLLECTION

Data entry and resolution will be performed in real-time concurrent with CPV data collection. All data will be linked to physicians via a unique identification number; only the study team will have access to their identities. Electronic data files will be password protected. Periodic audits will ensure data protection procedures are being followed. No physician will be mentioned by name in any data output.

8. STATISTICAL ANALYSIS

8.1 Determination of Sample Size

The study is adequately powered to detect differences in clinical practice patterns as measured by the CPVs. A sample size of 75 in each arm provides over 80% power to detect an estimated 6-7% difference in practice with alpha=0.05.

8.2 Planned Evaluations

Summary statistics will be determined for all variables. Simple cross tabulations will be prepared as part of the data resolution process. The calculation of basic descriptive statistics is used to check distributions and find outliers and out-of-range data. Numerical variables will be characterized in terms of means and standard deviations or by medians and interquartile range for skewed or highly non-normally distributed variables. For

hypothesis testing, tests will be conducted at a two-sided $\alpha = 0.05$. We have one primary outcome, quality as measured by the CPV.

Baseline Data Analysis

At baseline non-parametric estimates of differences will be calculated between the two groups (the control versus intervention groups to examine differences in CPV score and variance and utilization rates). Differences will be adjusted using multivariate and logistic regression models constructed to include age, gender, type of location (urban/suburban vs. rural), patients seen per week, practice size, single or multispecialty practice, practice ownership, and other practice characteristics and by patient characteristics such as type of payers, and use of biologics. Non-parametric estimates of differences in care utilization will be calculated and adjusted for physician, practice and patient characteristics described above.

Summary tables for each group will be generated for the following:

- Percent male
- Percent in single specialty practice
- Percent affiliated with an academic appointment
- Practice location
- Practice size (small, medium or large)
- Average days of week in practice
    - 1 to 2 days
    - 3 to 4 days
    - 5+ days
- Percent practices owned by
    - Physician group
    - Hospital
    - HMO
    - Community health center
    - Other
- Percent of patients covered by
    - Medicare
    - Commercial/Private
    - Medicaid
    - Self-pay / Uninsured
    - Other
- Mean (+/- 2 SD)
    - Years since fellowship
    - Number of patients with DD/ID seen per week
    - Age

<u>Dependent/Outcome Variables:</u>

- Behavioral
    - CPV® vignette score (overall and by domain)
    - Composite CPV diagnosis and treatment score

Main explanatory variables:

- FirstStep^{Dx} PLUS use
- FirstStep^{Dx} PLUS and NextStep^{Dx} PLUS use
- CPV® scores

Controlling for:

- Age
- Gender
- Number of years since fellowship
- Awareness of FirstStep^{Dx} PLUS and/or NextStep^{Dx} PLUS
- Practice location
- Practice size
- Type of location (urban/suburban vs. rural)
- Number of pediatric patients seen per week and number of DD/ID/ASD patients seen per week
- Number of physicians in practice
- Single or multispecialty practice
- Practice ownership (e.g., physician-physician group, community health center, etc.)
- Avg. type of coverage of patients (e.g., Medicare, Medicaid, etc.)

Longitudinal Data Analysis

With longitudinal data we can estimate the effect of FirstStep^{Dx} PLUS testing or the sequential use of FirstStep^{Dx} PLUS and NextStep^{Dx} PLUS testing on our primary and secondary outcomes. After the round 2 data is collected, we will conduct the longitudinal analysis incorporating type of intervention, time (before versus after), and the primary predictor, the time by intervention interaction (to look for differential changes over time). This allows a difference in difference analysis comparing differences between groups before and after intervention to assess the effect of FirstStep^{Dx} PLUS testing or the sequential use of FirstStep^{Dx} PLUS and NextStep^{Dx} PLUS testing.

Primary Outcome Variable:

- Quality of care (CPV® diagnosis/treatment score)

Secondary Outcome Variables:

- Use of appropriate therapy
- Utilization counts of laboratory tests and imaging studies ordered
- Overall CPV score

We will control for all potential confounders, including**:**

- Age
- Gender

- Number of years since fellowship
- Awareness of FirstStep[Dx] PLUS and/or NextStep[Dx] PLUS
- Practice location
- Type of location (urban/suburban vs. rural)
- Number of patients seen per week and number of DD/ID patients seen per week
- Number of physicians in practice
- Single or multispecialty practice
- Practice ownership (e.g. physician-physician group, community health center, etc)
- Avg. type of coverage of patients (Medicare, Medicaid, commercial, etc)

We will model CPV™ score as a continuous outcome variable in a multiple linear regression model, which controls for all potential confounders listed above. Our regression model is as follows, where CPV™ is the score for each physician, INTERV indicates type of intervention group and TIME indicates time period (pre and post-intervention).

$$Y_{it} = \text{CPVscore at time } t, \text{ physician } i$$
$$Y_{it} \sim Normal(\mu_{ijt}, \sigma_e^2)$$
$$\mu_{it} = \beta_0 + \beta_1 \text{TIME} + \beta_2 \text{INTERV} + \beta_3 \text{TIME} \times \text{INTERV} + \text{control variables}$$

For binary outcome variables, for example use of biologic therapy, we will use a logistic regression model. Our regression is as follows, where INTERV indicates type of intervention group and TIME indicates time period (pre and post-intervention). For outcomes with a Poisson distribution, the distributional assumption would be changed from Bernoulli to Poisson and the link would be changed from logit to log. Otherwise, the basic modeling and analysis strategy will remain the same.

$$Y_{it} = \text{ appropriate therapy } t, physician\ i$$
$$p_{it} = \text{ probability of an outcome for time } t, physician\ i$$
$$\text{logit}(E[Y_{it}]) = \beta_0 + \beta_1 \text{TIME} + \beta_2 \text{INTERV} + \beta_3 \text{TIME} \times \text{INTERV} + \text{control variables}$$

For count outcome variables such as number of follow-up visits we will use log linear regression (i.e., using a log link in the analysis). Cluster re-sampled bootstrapping will be used to check model distributional assumptions.

9. ADMINISTRATIVE CONSIDERATIONS

9.1 Study Compliance

The study will be conducted in compliance with this protocol, principles of ICH-6, GCP and the Declaration of Helsinki and all applicable national regulations governing clinical trials.

9.2 Protected Subject Health Information

A copy of the IRB approved informed consent may be audited. The investigator or designee **must** explain to each subject the purpose and nature of the study, the study procedures, the possible adverse effects and all other elements of consent as defined in 21CFR §50.

Confidential Proprietary Property of QURE Healthcare, LLC and Lineagen, Inc.

In accordance to individual local and national subject privacy regulations, the investigator or designee **must** explain to each subject prior to screening that for the evaluation of study results, the subject's protected information obtained during the study may be shared with Lineagen and its designees, regulatory agencies and IECs/IRBs. Lineagen will not use the subject's protected information or disclose it to a third party without applicable subject authorization. It is the investigator's or designee's responsibility to obtain a written permission to use protected information from each subject. Any data collected from a subject prior to withdrawal will not be used in the analysis of study results.

9.3 Retention of Records

The files of study subjects shall be retained in accordance with national legislation and the maximum period of time permitted by Lineagen. Lineagen will maintain records and documents on-site.

9.4 Confidentiality and Publication Policy

For publications, authorship will be determined according to the generally accepted principles of authorships and by mutual agreement.

REFERENCES

Boyle CA, Boulet S, Schieve LA, Cohen RA, Blumberg SJ, Yeargin-Allsopp M, et al. Trends in the Prevalence of Developmental Disabilities in US Children, 1997–2008. Pediatrics [Internet]. 2011 May 23 [cited 2014 Jun 6];peds.2010–2989. Available from: http://pediatrics.aappublications.org/content/early/2011/05/19/peds.2010-2989

Coulter ME, Miller DT, Harris DJ, Hawley P, Picker J, Roberts AE, Sobeih MM, Irons M. Chromosomal microarray testing influences medical management. Genet Med. 2011 Sep;13(9):770-6.

Ellison JW, Ravnan JB, Rosenfeld JA, Morton SA, Neill NJ, Williams MS, Lewis J, Torchia BS, Walker C, Traylor RN, Moles K, Miller E, Lantz J, Valentin C, Minier SL, Leiser K, Powell BR, Wilks TM, Shaffer LG. Clinical utility of chromosomal microarray analysis. Pediatrics. 2012 Nov;130(5):e1085-95.

Manning M, Hudgins L. Array-based technology and recommendations for utilization in medical genetics practice for detection of chromosomal abnormalities. Genet Med [Internet]. 2010 Nov [cited 2014 Jun 6];12(11):742–5. Available from: http://www.ncbi.nlm.nih.gov/pmc/articles/PMC3111046/

Matsunami N, Hadley D, Hensel CH, Christensen GB, Kim C, Frackelton E, Thomas K, da Silva RP, Stevens J, Baird L, Otterud B, Ho K, Varvil T, Leppert T, Lambert CG, Leppert M, Hakonarson H. Identification of rare recurrent copy number variants in high-risk autism families and their prevalence in a large ASD population. PLoS One. 2013;8(1):e52239

Matsunami N, Hensel CH, Baird L, Stevens J, Otterud B, Leppert T, Varvil T, Hadley D, Glessner JT, Pellegrino R, Kim C, Thomas K, Wang F, Otieno FG, Ho K, Christensen GB, Li D, Prekeris R, Lambert CG, Hakonarson H, Leppert MF. Identification of rare DNA sequence variants in high-risk autism families and their prevalence in a large case/control population. Mol Autism. 2014 Jan 27;5(1):5

Michelson DJ, Shevell MI, Sherr EH, Moeschler JB, Gropman AL, Ashwal S. Evidence report: Genetic and metabolic testing on children with global developmental delay: report of the Quality Standards Subcommittee of the American Academy of Neurology and the Practice Committee of the Child Neurology Society. Neurology. 2011 Oct 25;77(17):1629-35.

Peabody JW, Luck J, Glassman P, Jain S, Hansen J, Spell M, et al. Measuring the quality of physician practice by using clinical vignettes: a prospective validation study. Ann Intern Med. 2004 Nov 16;141(10):771–80.

Peabody JW, Strand V, Shimkhada R, Lee R, Chernoff D. Impact of Rheumatoid Arthritis Disease Activity Test on Clinical Practice. PLoS ONE. 2013 May 7;8(5):e63215.

Ronald A, Hoekstra RA. Autism spectrum disorders and autistic traits: A decade of new twin studies. Am J Med Genet [Internet]. 2011 Apr 1 [cited 2014 Jun 9];156(3):255–74. Available from: http://onlinelibrary.wiley.com/doi/10.1002/ajmg.b.31159/abstract

Shen Y, Dies KA, Holm IA, Bridgemohan C, Sobeih MM, Caronna EB, Miller KJ, Frazier JA, Silverstein I, Picker J, Weissman L, Raffalli P, Jeste S, Demmer LA, Peters HK, Brewster SJ, Kowalczyk SJ, Rosen-Sheidley B, McGowan C, Duda AW 3rd, Lincoln SA, Lowe KR, Schonwald A, Robbins M, Hisama F, Wolff R, Becker R, Nasir R, Urion DK, Milunsky JM, Rappaport L, Gusella JF, Walsh CA, Wu BL, Miller DT; Autism Consortium Clinical Genetics/DNA Diagnostics Collaboration. Clinical genetic testing for patients with autism spectrum disorders. Pediatrics. 2010 Apr;125(4):e727-35.

Volkmar F, Siegel M, Woodbury-Smith M, King B, McCracken J, State M; American Academy of Child and Adolescent Psychiatry (AACAP) Committee on Quality Issues (CQI). Practice parameter for the assessment and treatment of children and adolescents with autism spectrum disorder. J Am Acad Child Adolesc Psychiatry. 2014 Feb;53(2):237-57.
