# Supplementary material for: Clinical Utility of a Comprehensive, Whole Genome CMA Testing Platform in Pediatrics: A Prospective Randomized Controlled Trial of Simulated Patients in Physician Practices
Source: PLoS One. 2016 Dec 30;11(12):e0169064. doi: 10.1371/journal.pone.0169064 (PMC5201278; doi:10.1371/journal.pone.0169064)
Supplement: S1 File — (PDF) [file pone.0169064.s004.pdf]

CHESAPEAKE IRB

Human Research Protection Experts
IRB Services • Consultation • Education
6940 Columbia Gateway Drive, Suite 110
Columbia, MD 21046
410-884-2900

PROTOCOL APPROVAL WITH MODIFICATIONS

DATE: 20 Jun 2014

TO: John Peabody, MD PhD
QURE Healthcare

PROTOCOL: Lineagen, Inc. - 01-LIN-14, Establishing the Clinical Utility (ECU) of First StepDx PLUS and NextStepDx PLUS Study (Pro00009975)

APPROVAL DATE: 16 Jun 2014

EXPIRATION DATE: 16 Jun 2015

IRB APPROVED DOCUMENTATION:

Protocol Version:
- Clinical Study Protocol 01--□LIN--□2014 (Revision C, Dated 11JUNE14)

Consent Form:
- Peabody Pro00009975 Jun2014

Recruitment Material:
- Dear Doctor Letter (Not Dated)

Other Materials:
- Case Matrix for Lineagen CPV Vignettes (Draft 06.12.2014)
- Physician Questionnaire (Not Dated)

The IRB approved the above referenced protocol with the modifications listed below on 16 Jun 2014:

- **Modifications to the Informed Consent Form**
- **Due to this potential conflict of interest, Dr. Paul will not be involved in the informed consent process or recruitment for this study.**

If you wish to have the IRB reconsider the imposed modifications, you may follow the procedures outlined below:

1. Submit supporting documentation that addresses the IRB's concerns.
2. Provide a written justification for relief of any IRB imposed condition.

Please review the Investigator Handbook by accessing CIRBI™ (www.cirbi.net). Log on to your CIRBI homepage ("My Home") and select the "Reference Materials" tab for IRB requirements and guidance. A copy of the most recent IRB roster is also available under "Reference Materials".

Thank you for selecting Chesapeake IRB to provide oversight for your research project.

This document was printed from CIRBI ™ on 6/20/2014 4:31 PM
